# How right hemisphere damage after stroke can impair speech comprehension

**DOI:** 10.1093/brain/awy270

**Published:** 2018-11-09

**Authors:** Andrea Gajardo-Vidal, Diego L Lorca-Puls, Thomas M H Hope, Oiwi Parker Jones, Mohamed L Seghier, Susan Prejawa, Jennifer T Crinion, Alex P Leff, David W Green, Cathy J Price

**Affiliations:** 1 Wellcome Centre for Human Neuroimaging, UCL Queen Square Institute of Neurology, London, UK; 2 Faculty of Health Sciences, Universidad del Desarrollo, Concepcion, Chile; 3 FMRIB Centre, University of Oxford, Oxford, UK; 4 Cognitive Neuroimaging Unit, Emirates College for Advanced Education, Abu Dhabi, UAE; 5 Department of Neurology, Max Planck Institute for Human Cognitive and Brain Sciences, Leipzig, Germany; 6 Institute of Cognitive Neuroscience, University College London, London, UK; 7 Department of Brain Repair and Rehabilitation, UCL Queen Square Institute of Neurology, London, UK; 8 Experimental Psychology, Faculty of Brain Sciences, University College London, London, UK

**Keywords:** right-hemisphere stroke, lesion-deficit mapping, functional MRI, sentence comprehension, working memory

## Abstract

Acquired language disorders after stroke are strongly associated with left hemisphere damage. When language difficulties are observed in the context of right hemisphere strokes, patients are usually considered to have atypical functional anatomy. By systematically integrating behavioural and lesion data from brain damaged patients with functional MRI data from neurologically normal participants, we investigated when and why right hemisphere strokes cause language disorders. Experiment 1 studied right-handed patients with unilateral strokes that damaged the right (*n* = 109) or left (*n* = 369) hemispheres. The most frequently impaired language task was: auditory sentence-to-picture matching after right hemisphere strokes; and spoken picture description after left hemisphere strokes. For those with auditory sentence-to-picture matching impairments after right hemisphere strokes, the majority (*n* = 9) had normal performance on tests of perceptual (visual or auditory) and linguistic (semantic, phonological or syntactic) processing. Experiment 2 found that these nine patients had significantly more damage to dorsal parts of the superior longitudinal fasciculus and the right inferior frontal sulcus compared to 75 other patients who also had right hemisphere strokes but were not impaired on the auditory sentence-to-picture matching task. Damage to these right hemisphere regions caused long-term speech comprehension difficulties in 67% of patients. Experiments 3 and 4 used functional MRI in two groups of 25 neurologically normal individuals to show that within the regions identified by Experiment 2, the right inferior frontal sulcus was normally activated by (i) auditory sentence-to-picture matching; and (ii) one-back matching when the demands on linguistic and non-linguistic working memory were high. Together, these experiments demonstrate that the right inferior frontal cortex contributes to linguistic and non-linguistic working memory capacity (executive function) that is needed for normal speech comprehension. Our results link previously unrelated literatures on the role of the right inferior frontal cortex in executive processing and the role of executive processing in sentence comprehension; which in turn helps to explain why right inferior frontal activity has previously been reported to increase during recovery of language function after left hemisphere stroke. The clinical relevance of our findings is that the detrimental effect of right hemisphere strokes on language is (i) much greater than expected; (ii) frequently observed after damage to the right inferior frontal sulcus; (iii) task dependent; (iv) different to the type of impairments observed after left hemisphere strokes; and (v) can result in long-lasting deficits that are (vi) not the consequence of atypical language lateralization.

## Teaser

When right hemisphere strokes cause language impairments, it is generally assumed that the patients originally had atypical language lateralisation. Gajardo-Vidal *et al.* show how impaired speech comprehension can also arise when damage impinges on right hemisphere regions that are required to support speech comprehension even in the absence of atypical lateralisation.

**See Sheppard and Hillis (doi:10.1093/brain/awy291) for a scientific commentary on this article.**

## Introduction

Despite a long history of research favouring the view that the left hemisphere is dominant for language processing in most right-handed subjects, there is accumulating evidence that the right hemisphere contributes to (i) language function in neurologically normal individuals (Hartwigsen *et al.*, 2010*a*, *b*; Sollmann *et al.*, 2014); and (ii) language recovery after (a) left-hemisphere brain damage (Crinion and Price, 2005; Thiel *et al.*, 2006; Forkel *et al.*, 2014; Xing *et al.*, 2016; Nardo *et al.*, 2017); or (b) disruption of left-hemisphere processing (Hartwigsen *et al.*, 2013; Jung and Lambon Ralph, 2016). For example, studies of the neurologically normal brain suggest that bilateral inferior frontal and insula regions support the mapping from sound to lexical meaning (Bozic *et al.*, 2010), bilateral anterior temporal lobes are involved in the representation of conceptual knowledge (Rice *et al.*, 2015; Jung and Lambon Ralph, 2016; Lambon Ralph *et al.*, 2017) and bilateral inferior frontal and supramarginal gyri contribute to phonological decisions (Hartwigsen *et al.*, 2010*a*, *b*) and speech production (Sollmann *et al.*, 2014). For some language tasks, right-hemisphere activation may be driven by non-linguistic perceptual processing (Baumgaertner *et al.*, 2013), or the recruitment of attention and working memory (Vigneau *et al.*, 2011). It has also been argued that increased right inferior frontal activation in patients recovering from aphasia after left hemisphere stroke may be the result of upregulating non-linguistic cognitive processing (van Oers *et al.*, 2010) and the control of semantic retrieval (Thompson *et al.*, 2016, 2018).

The hypothesis that right hemisphere executive processing is necessary for normal language function contrasts with the dominant view that when language impairments are observed in right-handed patients with unilateral right hemisphere damage, they necessarily imply atypical language lateralization prior to the stroke (Marien *et al.*, 2004). However, the executive processing hypothesis is consistent with other literature that has associated right hemisphere activation with selective attention (Corbetta *et al.*, 2005; Hillis *et al.*, 2005; Bartolomeo *et al.*, 2012), imagery and domain general inhibitory control mechanisms (Aron *et al.*, 2004, 2014; Neef *et al.*, 2018) and working memory (Jonides *et al.*, 1993; Ravizza *et al.*, 2005).

Here we investigated whether a subset of impairments on language tasks after right hemisphere stroke in right-handed subjects can be explained by disruption to functions that are normally required for language. If so, we would expect the regions that are damaged in the language impaired patients to be activated when neurologically normal participants are performing the affected language tasks. Conversely, if impairments on language tasks after right hemisphere damage are always the consequence of atypical premorbid language processing in the right hemisphere then we would not expect the regions that are damaged in language impaired patients to be activated when right-handed neurologically normal participants are processing linguistic material. We would, however, expect that if patients have atypical language lateralization (e.g. at a semantic, phonological or syntactic level), then they would have impairments on all the tasks that tapped the affected language function. The evidence sought therefore rests on a combination of: (i) neuropsychological assessments of patients with right hemisphere damage to ascertain what level of processing is affected in those who have impaired performance on language tasks; (ii) lesion analyses to identify which right hemisphere brain regions were required pre-morbidly for the language tasks the patients had difficulty with; and (iii) functional neuroimaging studies of neurologically normal individuals to determine whether the right hemisphere regions damaged in the patients with language impairments are normally involved in language tasks; and whether their function is more consistent with linguistic or non-linguistic processing.

Rather than investigate all the different ways that language can break down after right hemisphere damage, the goal of Experiment 1 was to investigate the language processing impairment that was most frequently observed after right hemisphere strokes. This involved (i) identifying which language task from our standard assessment battery was most frequently impaired in 109 stroke survivors (all right-handed and native English speakers) who had unilateral right hemisphere damage; (ii) selecting a sample of patients that were impaired on this language task in the context of spared performance on other tasks (e.g. they were all able to recognize objects and match the semantic content of pictures); and (iii) considering what underlying processing deficit could explain why the selected patients were impaired on the identified language task. By including data from 369 right-handed patients with unilateral left hemisphere strokes, we were also able to compare the language task that was most frequently impaired after right hemisphere strokes to the language task that was most frequently impaired after left hemisphere strokes.

In Experiment 2, we investigated: (i) which right hemisphere regions were most frequently damaged in those patients with impaired performance on the language task identified in Experiment 1; and (ii) how frequently damage to these regions was observed in other patients who had right hemisphere lesions that did not impair performance on the language task identified in Experiment 1. This allowed us to establish whether the identified lesion sites were common or rare; and whether the effect of the lesion sites was typical or atypical.

In Experiment 3, we used functional MRI to define which parts of the regions identified in Experiment 2, if any, were activated when neurologically normal participants performed similar tasks to those used in Experiment 1. This allowed us to (i) pinpoint which parts of the identified lesion sites were actively involved in the most frequently impaired task; and (ii) reveal how these areas contribute to this task by studying how they respond in other language tasks. Finally, in Experiment 4, we investigated the function of the identified regions further by reporting a second functional MRI study of neurologically normal participants that examined how activation varied over a range of conditions that differed in their demands on linguistic and non-linguistic working memory.

By systematically integrating data from behavioural, lesion and functional imaging studies, we localize right hemisphere brain structures that support normal language; evaluate their linguistic or non-linguistic functions and offer an explanation for how right hemisphere stroke damage can impair language performance.

## Materials and methods

This study was approved by the London Queen Square Research Ethics Committee.

### Experiment 1: Which language task is most frequently impaired after right hemisphere stroke?

Data were extracted from the PLORAS database, which holds the results of language assessments and high resolution T_1_-weighted structural MRI brain scans from hundreds of stroke survivors recruited in the UK, months to years after their stroke (Seghier *et al.*, 2016). Language abilities in all patients were assessed using the Comprehensive Aphasia Test (Swinburn *et al.*, 2004), which uses standardized procedures, and T-scores, to classify each patient’s performance as normal or impaired on 27 different cognitive and language tasks (Table 1). Brain scans were high resolution T_1_-weighted MRI acquired with 176 sagittal slices and a matrix size of 256 × 224, yielding a final spatial resolution of 1 mm isotropic voxels.

**Table 1:** Incidence of impaired performance for all right and left hemisphere stroke patients in Experiment 1

The Comprehensive Aphasia Test (CAT)		**RH**	**LH**	**RH**	**LH**
**Sections**	**Subtests**	**Without VPI *n* = 93**	**Without VPI *n* = 307**	**With VPI *n* = 16**	**With VPI *n* = 62**
**I: The Cognitive Screen**					
	1. Line bisection	0	0	12	23
	2. Match pic-to-pic (semantic)	2 (2%)	6 (2%)	6	30
	3. Recognition memory	3 (3%)	10 (3%)	3	31
	Semantic memory Score^a^	0	0	7	44
	4. Word fluency	1 (1%)	90 (29%)	2	45
	5. Gesture object use	2 (2%)	42 (14%)	3	21
	6. Arithmetic	0	3 (1%)	1	11
**II: The Language Battery**					
Comprehension	7. Match aud word-to-pic	2 (2%)	54 (18%)	8	32
	8. Match auditory sentence-to-picture	12 (13%)	140 (46%)	9	57
	9. Match auditory paragraph	0	44 (14%)	2	22
	10. Match written word-to-picture	8 (9%)	91 (30%)	8	47
	11. Match written sentence-to-picture	2 (2%)	109 (36%)	10	47
Repetition	12. Repetition of heard words	5 (5%)	151 (49%)	6	39
	13. Repetition of complex words	4 (4%)	120 (39%)	4	35
	14. Repetition of pseudowords	7 (8%)	104 (34%)	6	39
	15. Repetition of digit strings	2 (2%)	125 (41%)	0	41
	16. Repetition of sentences	1 (1%)	137 (45%)	1	45
Spoken output	17. Naming objects	5 (5%)	146 (48%)	6	47
	18. Spoken picture description	7 (8%)	167 (54%)	11	52
Reading aloud	19. Reading words	3 (3%)	153 (50%)	8	44
	20. Reading complex words	2 (2%)	133 (43%)	6	39
	21. Reading function words	0	37 (12%)	0	22
	22. Reading pseudowords	1 (1%)	142 (46%)	5	45
Writing	23. Copying letters	3 (3%)	26 (8%)	4	26
	24. Written picture naming	0	61 (20%)	4	33
	25. Writing to dictation	4 (4%)	125 (41%)	6	44
	26. Written picture description	6 (6%)	154 (50%)	6	46

^a^Semantic memory score is a combined score from picture-to-picture semantic matching and recognition memory. The action naming task was not included in the table because of the high variability in scores across neurologically-normal controls (i.e. low specificity). The incidence of impaired performance is showed in absolute numbers and percentages.

LH = left hemisphere stroke patients; RH = right hemisphere stroke patients; VPI = visual perceptual impairments.

Patients for the current study were selected according to the following inclusion criteria: (i) unilateral stroke attested by a clinical neurologist (A.P.L.) and defined by an automated lesion identification algorithm (Seghier *et al.*, 2008); (ii) >1 cm^3^ of right hemisphere damage and <1 cm^3^ of left hemisphere damage or >1 cm^3^ of left hemisphere damage and <1 cm^3^ of right hemisphere damage; (iii) native speakers of English; (iv) right-handed prior to the stroke onset; and (v) tested >3 months and <10 years after their stroke. Our selection criteria did not consider the within hemisphere site of the lesion or the presence or absence of aphasia. These criteria were met by 109 patients with right hemisphere strokes and 369 patients with left hemisphere strokes (see Tables 1 and 2 for demographic, clinical and behavioural data). For each of these groups, we identified and compared the language task that was most frequently impaired. For the right hemisphere patients, we also investigated how consistently impairments on the most frequently affected task co-occurred with impairments on other tasks. This allowed us to generate hypotheses about the level of processing that was most likely to be affected; and to identify a subset of patients with consistent neuropsychological profiles that could be investigated further with lesion analyses.

**Table 2:** Demographic and clinical details for all right hemisphere and left hemisphere stroke patients

**Demographic and clinical details**		**Full sample**		**Without VPI**	
		**RH**	**LH**	**RH**	**LH**
		***n* = 109**	***n* = 369**	***n* = 93**	***n* = 307**
Age at scan, years	Mean	59.3	59.6	58.3	58.5
	SD	12.7	12.7	13.1	12.5
	Minimum	23.1	21.3	23.1	21.3
	Maximum	86.9	90.0	86.9	90.0
Years since stroke	Mean	3.3	3.3	3.2	3.3
	SD	2.3	2.5	2.1	2.4
	Minimum	0.3	0.3	0.3	0.3
	Maximum	9.2	10.0	9.2	10.0
Lesion size, cm^3^	Mean	63.4	76.0	49.0	67.8
	SD	81.7	82.7	63.8	75.2
	Minimum	1.1	1.0	1.1	1.0
	Maximum	356.3	427.5	276.3	385.2
Years of education	Mean	14.6	14.4	14.8	14.5
	SD	2.9	3.0	3.0	3.0
	Minimum	10.0	10.0	10.0	10.0
	Maximum	22.0	26.0	22.0	26.0
Gender, *n*	Females	41	105	34	89
	Males	68	264	59	218
Self-report assessment, *n*	Difficulty understanding in first year	37 (93)	214 (291)	35 (83)	177 (244)

Values in brackets indicate the number that had data on the self-assessment questionnaire available. VPI = visual perceptual impairments.

### Experiment 2: Which regions are damaged in the patients of interest from Experiment 1?

First, we used voxel-based morphometry (VBM; Ashburner and Friston, 2000), implemented in SPM12 with an unequal variance two sample *t*-test (Mummery *et al.*, 2000), to identify which regions were significantly more damaged in our patients of interest than in other (control) patients with right hemisphere strokes that did not affect performance on the task identified in Experiment 1. Details of these patient groups are provided in the ‘Results’ section. Second, we individually examined the lesion site in each patient with the impairment of interest to establish (i) how many had damage to the regions identified by VBM; and (ii) which regions were damaged in those who preserved the lesion sites identified by VBM. Third, in all other patients, we examined how frequently each of the regions of interest was damaged in the absence of an impairment on the task identified in Experiment 1.

As in Price *et al.* (2010), Gajardo-Vidal *et al.* (2018) and Lorca-Puls *et al.* (2018), the lesion images entered into the VBM analysis were continuous measurements of structural ‘abnormality’ at each and every voxel, calculated by comparing estimates of grey and white matter in each patient to a sample of 64 neurologically normal controls (Seghier *et al.*, 2008). The output is a 3D lesion image in standard MNI space, indicating the degree of structural abnormality on a continuous scale from 0 (completely normal) to 1 (completely abnormal) with a voxel size of 2 × 2 × 2 mm^3^. The advantages of using the fuzzy lesion images have been described in full elsewhere (Price *et al.*, 2010; Gajardo-Vidal *et al.*, 2018).

The analysis included lesion size as a covariate of no interest and the search volume was limited to voxels that were classified as lesioned in at least five patients (as in Fridriksson *et al.*, 2016; for rationale, see Sperber and Karnath, 2017). Given that large lesions are more likely to damage critical regions, the inclusion of lesion volume as a covariate of no interest in voxel-based analyses might have a negative impact on the identification of significant lesion-deficit associations (for more details, see Butler *et al.*, 2014). We therefore replicated the same analysis after removing the lesion volume regressor.

The presence of a lesion and lesion volume were based on binary lesion images generated by thresholding the fuzzy images. The threshold used to convert the fuzzy to binary images was 0.3 as recommended in Seghier *et al.* (2008). Each binary lesion image was visually inspected by the operator. The boundaries of the lesion may differ slightly from what is seen by eye but provide an objective rather than subjective measure of structural abnormality. There is no gold standard of true abnormality. As a result classification errors are treated as ‘noise’ in the analysis, biasing towards false negatives rather than false positives.

The statistical output from the comparison of brain structure in the group of interest versus the control group was thresholded at *P* < 0.05 after family-wise error (FWE) correction for multiple comparisons across the whole search volume (estimated using random field theory as implemented in SPM; Flandin and Friston, 2015). Having identified a significant lesion-deficit mapping, we then examined the extent of the effect at a voxel-level threshold of *P* < 0.001 uncorrected, *P* < 0.05 FWE-corrected cluster-level. All surviving voxels became our ‘VBM region’. Within this region, we report the *x*, *y*, *z* MNI co-ordinates corresponding to the peak *Z*-scores.

### Experiment 3: Are the regions identified in Experiment 2 involved in normal sentence comprehension?

Twenty-five neurologically normal, native English speakers who were all right-handed according to the Edinburgh Handedness Inventory (Oldfield, 1971), were included in this functional MRI study, which aimed to identify whether the right hemisphere regions from Experiment 2 were activated: (i) during the task of interest from Experiment 1 (i.e. auditory sentence-to-picture matching; Supplementary Fig. 2); and (ii) by conditions that varied demands on auditory, visual, phonological, semantic, sentence processing and verbal short-term memory.

There were 10 conditions that each presented stimuli comprising two pictures, auditory speech or both (Table 3) during semantic matching, auditory repetition or speech retrieval tasks. The 10 conditions contributed to three embedded factorial designs (Supplementary Fig. 1). The first factorial design (Design A) combined six task conditions to compare sentences to objects during: (i) speech-to-picture matching; (ii) auditory repetition; and/or (iii) speech retrieval. The second (Design B) combined four naming conditions to isolate sentence processing from the presence or absence of: (i) object names; and (ii) verbs. The third (Design C) compared semantic associations to speech production tasks in the auditory and visual modalities (four conditions) while keeping stimuli constant within modality and the task constant across modality. In this third design, the interaction of task and stimulus modality tests the demands on: (i) phonological (name) retrieval, which is greater for speech production than semantic decisions when the stimuli are pictures of objects than when the stimuli are heard object names (i.e. when the speech production task is picture naming rather than auditory repetition); and (ii) verbal short-term memory, which is greater for semantic decisions than speech production when the stimuli are auditory object names (which need to be held in memory while a semantic association is assessed) than when the stimuli are visually presented objects (that do not require auditory memory).

**Table 3:** Experimental design and second level contrasts for Experiment 3

**Paradigm details**					**Second level contrasts**									
**ID**	**Task name**	**Stimulus**		**Response**	**Design A Sentences > 2 Object names**			**Design B Sentences Obj/Verbs**			**Design C Stimulus/task vSTM/PhR**			
		**Hear**	**See**	**Finger/speech**	**Main effect**	**Inter**		**2 Obj**	**Verbs**	**Inter**	**Aud**	**Sem**	**SP**	**Inter**
1	Aud-Pic Match	Sent	Event	Finger	1	1	1	0	0	0	0	0	0	0
2	Aud-Pic Match	2 Obj	2 Obj	Finger	−1	−1	−1	0	0	0	0	0	0	0
3	Vis Sem Assoc	-	2 Obj	Finger	0	0	0	0	0	0	−1	1	−1	−1
4	Aud Sem Assoc	2 Obj	-	Finger	0	0	0	0	0	0	1	1	−1	1
5	Aud Rep Sent	Sent	-	SP sent	1	0	−1	0	0	0	0	0	0	0
6	Aud Rep 2 Obj	2 Obj	-	SP names	−1	0	1	0	0	0	1	−1	1	−1
7	Produce Sentence	-	Event	SP sent	1	−1	0	1	1	1	0	0	0	0
8	Name 2 Objects	-	2 Obj	SP names	−1	1	0	1	−1	−1	−1	−1	1	1
9	Produce Verb	-	Event	SP verbs	0	0	0	−1	1	−1	0	0	0	0
10	Name Colours	-	Pattern	SP colour	0	0	0	−1	−1	1	0	0	0	0

Details of each of the 10 tasks (illustrated in Supplementary Fig. 1) and the weighting that each task was given in the factorial analysis of the three embedded designs (Designs A–C; see ‘Materials and methods’ section).

2 Obj = pictures of two objects or two object names; Aud = auditory presentation of object names or sentences; Aud Rep = auditory repetition; Aud-Pic Match = matching an auditory stimulus to a picture; Inter = interaction between two effects; PhR = phonological retrieval (highest for object naming/sentence production); Sem Assoc = matching two objects according to whether they are semantically related or not; Sent = sentences; SP = speech production; Vis = visual presentation of pictures; vSTM = verbal short-term memory (highest for auditory semantic associations).

In Design A, Inter = effect of sentences > 2 Obj on Aud-Pic Match > other tasks. In Design B, Inter = sentences (object names and verbs) > object names or verbs. In Design C, Inter = vSTM or PhR.

Each of the 10 tasks was presented in a separate scanning session (counterbalanced across participants) with five blocks of four stimuli interleaved with 16.96 s of resting with eyes open (20 stimuli and 40 object concepts per condition). Experimental and participant details are provided in Table 5.

### Stimuli selection, creation and counterbalancing

Overall, we used a total of 120 object concepts that were easy to recognize and name when presented in picture format (using high definition pictures drawn by a professional artist). Each of the 120 objects was paired to three others making 3 × 60 = 180 pairs. The first pairing involved two objects interacting with one another to indicate an event, with a corresponding sentence (e.g. the cat is drinking from the jug). These were used for sentence production, sentence repetition, verb naming and auditory sentence-to-picture matching. The second pairing presented two unrelated objects (e.g. ‘car and plate’), that were used for object naming, auditory repetition, colour naming or auditory word to picture matching. The third pairing involved semantic pairs that were half related (e.g. ‘door and key’) and half unrelated (e.g. ‘deer and barrel’). The three different pairings resulted in a total of 180 different pairs (3 × 60). Stimulus repetitions, within subject, were avoided by repeating objects: (i) with a different pair; (ii) in different stimulus modalities (auditory versus visual or both); or (iii) with a change in task and response (matching versus spoken). Over participants each object was seen an equivalent number of times in each condition.

Compared to Experiment 1, auditory sentence-to-picture matching in Experiment 3 included: sentences with simpler structures (i.e. object-verb-subject) and only four possible actions/verbs (jumping, falling, eating or drinking). The limited number of verbs was to minimize intersubject variability in word choice (or structure) during production.

During sentence production, auditory repetition, object and colour naming, participants were instructed to speak aloud in the scanner so that we could distinguish correct and incorrect responses. Head movement was limited and corrected using unwarping during image realignment. The degree of movement made by each participant was monitored and it was not necessary to remove any participants due to excessive movement-related artefacts.

### Functional MRI data acquisition and analysis

Functional MRI data were acquired on a 3 T Trio scanner (Siemens Medical Systems) using a 12-channel head coil and a gradient-echo EPI sequence with 3 × 3 mm in-plane resolution (repetition time/echo time/flip angle: 3080 ms/30 ms/90°, extended field of view = 192 mm, matrix size = 64 × 64, 44 slices, slice thickness = 2 mm, and interslice gap = 1 mm). Anatomical data were high resolution T_1_-weighted structural images, acquired using exactly the same scanning parameters as for the patients imaged on the 3 T scanner (see above).

Data preprocessing and statistical analyses were performed with the Statistical Parametric Mapping (SPM12) software package (Wellcome Centre for Human Neuroimaging, London UK; http://www.fil.ion.ucl.ac.uk/spm/). All functional volumes were spatially realigned, unwarped, normalized to MNI space using the new unified normalization-segmentation procedure, and smoothed with a 6 mm full-width half-maximum isotropic Gaussian kernel, with a resulting voxel size of 3 × 3 × 3 mm.

### First-level analyses

Each preprocessed functional volume was entered into a subject-specific, fixed-effect analysis using the general linear model (Friston *et al.*, 1995). All stimulus onset times, for all conditions, were modelled as single events (Mechelli *et al.*, 2003). Stimuli with correct responses were modelled separately from stimuli with incorrect responses. Stimulus functions were convolved with a canonical haemodynamic response function. To exclude low-frequency confounds, the data were high-pass filtered using a set of discrete cosine basis functions with a cut-off period of 128 s. The contrasts of interest were generated for each of the conditions (correct trials only) relative to fixation baseline.

### Second-level analyses

The contrasts from the first level analysis—one for each task relative to rest—were entered into a 2 × 10 repeated measures ANOVA. The second variable was intertrial interval, which was 5 s for 12 subjects and 7 s for 13 subjects. This was entered as a between-subjects factor with the 10 tasks as a within-subjects factor. As there were no regions within the search volume showing a main effect of intertrial interval (5 s and 7 s intertrial interval groups) nor Intertrial interval × Task interactions, our results report the mean across both groups. The statistical threshold for second level contrasts was corrected for multiple comparisons (i.e. family-wise error correction, FWE) within the lesion sites identified in Experiment 2 (Fig. 2A). Peak co-ordinates (*x, y, z*) are reported in MNI space.

### Experiment 4: Are the regions identified in Experiment 3 involved in non-linguistic working memory?

This functional MRI study investigated whether the right hemisphere regions associated with the condition/task of interest in Experiments 2 and 3, were activated when a new set of 25 neurologically normal, right-handed, native English speakers were making one-back matching decisions (i.e. is the stimulus currently being displayed the same as the previously presented stimulus?) on linguistic and non-linguistic stimuli. There were eight different conditions in a 2 × 2 × 2 factorial design that manipulated: (i) auditory versus visual stimuli; with (ii) speech versus non-speech content; and (iii) high versus low semantic content (Table 4). In addition, the experimental paradigm included eight corresponding speech production conditions that are not relevant to the current study but have been reported in Oberhuber *et al.* (2016), which investigated phonological processing in the left supramarginal gyrus. Condition order was fully counterbalanced. Experimental and participant details are provided in Table 5.

**Table 4:** Experimental design and second level contrasts for Experiment 4

**Paradigm details**		**Second level contrasts**							
**ID**	**Task**	**Stimulus Modality**		**Semantic content^a^ (semantic)**		**Sublexical phonology (phonology)**		**Interaction (semantic and phonology)**	
		**Visual**	**Auditory**	**Present**	**Absent**	**Present**	**Absent**	**Present**	**Absent**
O	See pictures of objects	1	−1	1	−1	−1	1	−1	1
W	See written object names	1	−1	1	−1	1	−1	1	−1
P	See written pseudowords	1	−1	−1	1	1	−1	−1	1
B	See coloured patterns	1	−1	−1	1	−1	1	1	−1
O	Heard sounds of objects	−1	1	1	−1	−1	1	−1	1
W	Heard names of objects	−1	1	1	−1	1	−1	1	−1
P	Heard pseudowords	−1	1	−1	1	1	−1	−1	1
B	Heard male/female voice	−1	1	−1	1	−1	1	1	−1

There were eight one-back matching with finger press response tasks that factorially manipulated the presence or absence of semantic content, the presence or absence of sublexical phonology, using heard or written pseudowords (P), words (W) objects (O) or baselines (B). See text for details.

^a^Task/condition of interest.

**Table 5:** Experimental details for Experiments 3 and 4

**Partcipants**	**Experiment 3**	**Experiment 4**
*n*	25	25
Gender, *n*, females/males	15/10	12/13
Mean age in years (±SD)	30.4 (3.9)	31.4 (5.9)
**Timing parameters**		
Stimulus duration, s		
Visual stimuli	2.5	1.5
Auditory stimuli/words**^a^**	1.8–2.5	0.64
Auditory pseudowords	-	0.68
Intertrial interval, s**^b^**	5/7	2.5
Block length, s**^c^**	20/28	22.5
Total time for each run, min**^d^**	3.4/4.1	3.2
Total acquisition time, min	33.9/41.1	51.2
Number of stimuli per block	4	9 (incl. one repeat)
Number of blocks per run	5	4
Total number of stimuli per run	20	36
Number of runs	10	16
**Scanning parameters**		
Repetition time, s	3.1	3.1
Number of slices	44	44
Number of volumes per run	61/85	62
Number of dummy acquisitions	5	5

^a^For Experiment 3, auditory stimuli included single words and sentences whereas for Experiment 4 auditory stimuli included single words only.

^b^For Experiment 3, 5 s intertrial interval = 5 s intertrial interval group (*n* = 12), 7 s intertrial interval = 7 s intertrial interval group (*n* = 13).

^c^For both functioanl MRI experiments, each block began with instructions for 3.1 s.

^d^For Experiment 3, each run ended with a resting period of 16.96/18.2 s for 5 s/7 s intertrial interval, respectively. For Experiment 4, each run ended with a resting period of 16 s.

The functional MRI acquisition, preprocessing and first-level analyses were exactly the same as described above for Experiment 3. In the second-level analysis, we used a one-way ANOVA, with eight contrasts, one for each one-back condition relative to rest and report the main effect of semantic content (words and objects versus pseudowords and meaningless baselines), sublexical phonological cues (words/pseudowords versus pictures and non-verbal sounds), the interactions between these variables and with stimulus modality (visual versus auditory). The search volume was restricted to only include voxels that were part of two spheres (radius of 3 mm) centred on the peak co-ordinates obtained from the contrast of interest in Experiment 3. We report effects that survived a voxel-level threshold of *P* < 0.05, after FWE-correction for multiple comparisons within the regions of interest (Fig. 2B). Peak co-ordinates (*x, y, z*) are reported in MNI space.

### Data availability

The data that support the findings of this study are available from the senior author (c.j.price@ucl.ac.uk) upon reasonable request.

## Results

### Experiment 1: Which language task is most frequently impaired after right hemisphere strokes?

The language task that was most frequently impaired after right hemisphere stroke damage was auditory sentence-to-picture matching (Table 1 and Supplementary Table 3). Even after excluding all patients with visual perceptual deficits, the incidence of impairments on the auditory sentence-to-picture matching task was 13% (12/93) compared to 0–9% (mean = 4%) on all other language tasks (Table 1). In contrast, in patients with left hemisphere stroke damage the most frequently affected task (in those who did not have visual perceptual impairments) was spoken picture description, with an incidence of 54% (167/307) compared to: 1–50% (mean = 30%) on all other tasks and 46% (140/307) on auditory sentence-to-picture matching. The auditory sentence-to-picture matching and spoken picture description tasks are therefore the most sensitive language comprehension and production indices, respectively, in our language assessment.

To characterize differences in performance between language comprehension (i.e. auditory sentence-to-picture matching task) and production (i.e. spoken picture description task) in left-hemisphere versus right-hemisphere stroke patients further, a 2 × 2 mixed factorial ANOVA was conducted on task scores with Task (Production versus Comprehension) as a within-subjects factor and Hemisphere Damaged (Left versus Right) as a between-subjects factor. We found a main effect of Hemisphere Damaged [*F*(1,398) = 87.70, *P* < 0.001], indicating that left-hemisphere stroke patients (mean = 58.9) performed, on average, significantly worse than right-hemisphere stroke patients (mean = 66.9). The main effect of Task was not significant [*F*(1,398) = 2.48, *P* = 0.116] but there was a significant Hemisphere × Task interaction [*F*(1,398) = 11.26, *P* = 0.001]. *Post hoc* tests confirmed that patients with unilateral right-hemisphere lesions had poorer language comprehension (mean = 65.8) than production (mean = 68.0; *P* = 0.005), while a trend in the opposite direction was observed in patients with unilateral left-hemisphere lesions (mean Comprehension = 59.3 versus Production = 58.6; *P* = 0.066).

To investigate the processing level that was affected in the 12 patients with right hemisphere damage and impaired scores on the auditory sentence-to-picture matching task, we considered how these patients performed on other language tasks. We found that 9/12 of these patients were able to: (i) repeat heard pseudowords and digit strings (two classic tests of speech perception and phonological working memory); and (ii) match visual sentences to pictures (that placed the same level of demand on semantic, syntactic and picture processing as auditory sentence-to-picture matching) (Supplementary Tables 1 and 2). They therefore had impairments in auditory sentence-to-picture matching that could not be explained by difficulties with speech perception, semantics, phonological working memory, syntactic processing, or the integration of the syntactic structure of a sentence with semantic information.

Examination of the frequency and type of errors that these nine patients made during auditory sentence-to-picture matching (Table 6) indicated that they had impaired sentence comprehension: all nine made at least two errors (either incorrect responses or delays/self-corrections) on the 10 trials presenting difficult, reversible sentences (e.g. ‘The singer hits the soldier’), 7/9 made errors on the sentences with the simplest structures (e.g. ‘The woman is drinking’) and 6/9 reported that their auditory speech comprehension had been compromised by their stroke (see Table 2 for the results of our self-report questionnaire that was conducted without the patient’s knowledge of the test results).

**Table 6:** Type of errors made by the nine patients of interest in the auditory sentence-to-picture matching task

**Type of sentences**	**Patient ID**									**Stimulus location**
	**1**	**2**	**3**	**4**	**5**	**6**	**7**	**8**	**9**	
**Non-reversible sentences**									
1. The woman is drinking	-	-	1	-	-	0	-	-	1	RU
2. The man is walking		1	-	-	-	1	0	1	-	LB
3. She is laughing	-	-	-		-	-	-	-	-	LB
4. The man is eating the apple	-	-	-	-	-	-	-	-		RB
5. The woman is painting the wall	-	-	-	1	-	-	1	-	-	RU
6. The dog is sitting on the table	-	-	-	-	-	-	-	-	-	LU
**Reversible sentences**										
7. The apple is under the shoe	-	-	-	-	-	-	-	-	-	RU
8. The nurse shoots the butcher	0	1	-	0	1	-	-	-	-	RU
9. The singer hits the soldier	0	0	0	0	0	1	0	0	0	LB
10. The policeman is painted by the dancer	-	-	-	-	-	0	1	-	0	LU
11. The butcher is chased by the nurse	-	-	-	-	0	1	-	-	-	RB
12. The dancer paints the policeman	-	-	-	-	-	-	-	-	-	LU
13. The shoe under the pencil is blue	0	0	0	1	1	-	1	0	1	RB
14. The carpet the cat is on is red	-	-	1	-	-	-	-	-	-	LU
15. The red pencil is under the shoe	-	-	-	-	-	-	-	-	-	LB
16. The flower in the cup is blue	-	-	-	-	-	-	-	-	-	RB

Type of errors made by the nine patients with impairments on the auditory sentence-to-picture matching task. 1 denotes a score of 1 for an accurate but delayed response, repetition of the target by the examiner and/or self-correction. 0 denotes a score of 0 for incorrect responses. All other trials had a score of 2. Importantly, all incorrect responses on reversible sentences corresponded to instances where the subject-verb-object relationship was reversed. For example, in Sentence 13, patients chose alternative (C): The pencil under the shoe is blue. The last column shows the location in which the target sentences were displayed: R/L = right/left; B/U = bottom/upper. Patients had to select a picture, from a set of four (2 × 2 array) that best illustrated the sentence that they heard. Patients 1–9 refer to the following IDs in the PLORAS database: PS0316, PS0383, PS0448, PS0670, PS0870, PS1172, PS1211, PS1550 and PS2627, respectively.

Below, we systematically investigate whether their speech comprehension impairments might be the consequence of a reduction in the overall processing capacity available for syntactic, interpretive, and task-related operations (Caplan *et al.*, 2007), particularly in the auditory modality (Thompson and Jefferies, 2013). This involved identifying which right hemisphere regions were damaged in the nine patients of interest with impairments in auditory sentence-to-picture matching and how these regions respond to auditory and visual linguistic and non-linguistic processing in neurologically normal individuals (Experiments 3 and 4).

### Experiment 2: Which regions are damaged in the patients of interest from Experiment 1?

**Figure 1:**
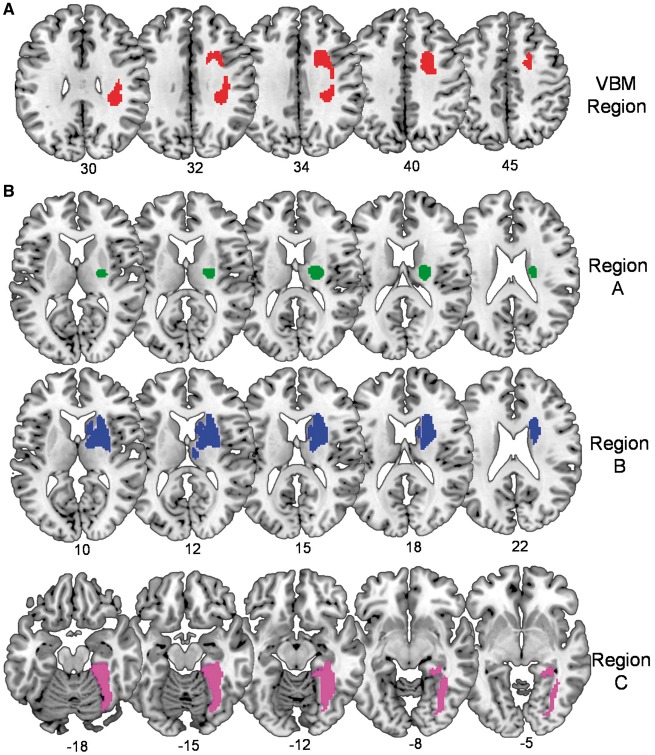
**Right hemisphere lesion sites associated with impaired auditory sentence-to-picture matching. (A)** The *top row* shows the region identified in our voxel-based lesion-deficit analysis (VBM region in Experiment 2). (**B**) The *bottom rows* show the ‘small lesions’ A, B and C from three patients with impaired auditory sentence-to-picture matching and no damage to the VBM region. Numbers below indicate the corresponding MNI coordinates.

The voxel-based lesion-deficit analysis compared the lesion sites in our nine patients of interest to those of 75 control patients who did not have auditory sentence-to-picture matching impairments. This yielded one significant cluster (with 782 contiguous voxels) centred on the dorsal aspect of the right superior longitudinal fasciculus (peak Z-score = 6.7 at +22, +8, +40) and impinging on the right inferior frontal sulcus (Z-score = 3.3 at +32, +4, +34) (Fig. 1A). When the analysis was replicated without including lesion volume as a covariate of no-interest, virtually the same lesion-deficit associations were identified. Henceforth, we focus on the results of the VBM analysis that factored out linear effects from lesion size.

*Post hoc* analyses found that the ‘VBM region’ (i.e. 782 voxels in size) was substantially damaged (>70%) in 6/9 of the patients of interest (67%) but only 3/75 (4%) of the control patients. Put the other way, nine patients had >70% damage to the VBM region and 6/9 (67%) of these patients had auditory sentence-to-picture matching impairments. In the remaining three patients of interest, the VBM region was completely preserved (0% damage). These three patients all had relatively small lesions affecting parts of the putamen, thalamus, caudate or right temporal lobe; see ‘small lesions’ A, B and C in Fig. 1B. Examination of how these regions were damaged across the two samples (patients of interest and control patients) indicated that small region A was >95% damaged in 3/9 patients of interest and in 14/75 control patients. Put the other way, 17 patients had >95% damage to region A and 3/17 (18%) of these patients had auditory sentence-to-picture matching impairments. Small region B was >95% damaged in 3/9 patients of interest and 5/75 control patients. Put the other way, eight patients had >95% damage to region B and 3/8 (38%) of these patients had auditory sentence-to-picture matching impairments. Small region C was rare and only damaged in the patient of interest defining it.

### Experiment 3: Are the regions identified in Experiment 2 involved in normal sentence comprehension?

**Figure 2:**
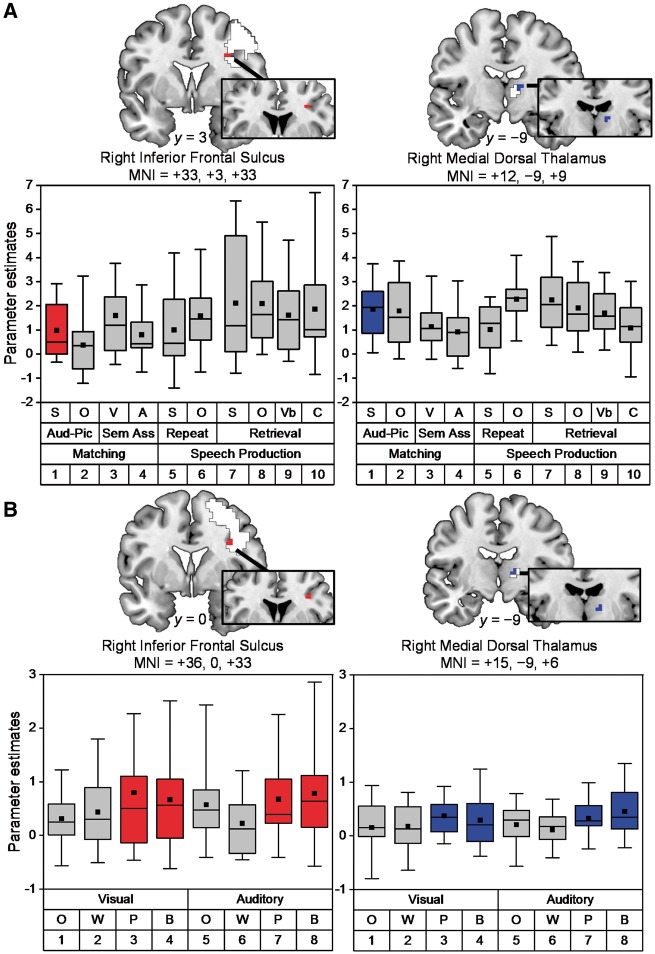
**Illustration of right hemisphere activation in Experiments 3 and 4.** (**A**) *Top row*: coronal slices show peak activations for auditory sentence-to-picture matching relative to rest in the right inferior frontal sulcus (in red) and the right medial dorsal thalamus (in blue) at coordinates [*x* = +33, *y* = +3, *z* = +3] and [*x* = +12, *y* = −9, *z* = +9], respectively. White regions show the full extent of activation, after FWE correction for multiple comparisons across the whole brain. Box plots depict medians with interquartile ranges and whiskers represent the 5th and 95th percentiles. The black squares indicate the mean value for each task. Aud-Pic = auditory-to-picture matching tasks; C = colour naming; S and O = sentences and objects; Sem Ass = semantic association tasks; V and A = visual and auditory presentation; Vb = verb (action) naming. Numbers (*bottom row*) = condition number (Table 3 and Supplementary Fig. 1). (**B**) *Bottom row*: coronal slices showing peak activations for one-back matching on pseudowords (P) and baselines (B) more than words (W) or pictures of objects (O) in the right inferior frontal sulcus (five voxels in red) and the right medial dorsal thalamus (five voxels in blue) at coordinates [*x* = +36, *y* = 0, *z* = +33], Z score = 3.9, *P*_FWE-corr_ = 0.001, and [*x* = +15, *y* = − 9, *z* = +6], Z score = 3.8, *P*_FWE-corr_ = 0.001, respectively. White regions show the full extent of activation from whole brain analysis; *P* < 0.001, uncorrected. Box plots depict medians with interquartile ranges and whiskers represent the 5th and 95th percentiles. The black squares indicate the mean value for each task.

Within the region of interest (encompassing the VBM region and small regions A and B from Experiment 2), we found significant activation for auditory sentence-to-picture matching (relative to rest) in the right inferior frontal sulcus (peak Z-score = 6.3 at +33, +3, +33), and the right mediodorsal thalamus (peak Z-score = 4.3 at +12, −9, +9) (Fig. 2A). The same regions were also activated during all other conditions (Fig. 2A) with no significant differences (*P* > 0.001 uncorrected) in these regions for any of the effects of interest (Table 3) including: sentences compared to objects during any of the three task manipulations (Design A); sentences, verbs or object names (Design B); or auditory stimuli, semantic associations, verbal short-term memory, speech production or phonological (name) retrieval (Design C). Nor were there any significant effects for the reverse contrasts.

In summary, the results of Experiment 3 provide evidence that parts of the right hemisphere regions that were damaged in patients with auditory sentence-to-picture matching impairments (Experiment 2) are activated when neurologically normal participants are matching auditory sentences to pictures but there was no evidence that these regions were performing exclusively linguistic functions.

### Experiment 4: Are the regions identified in Experiment 3 involved in non-linguistic working memory?

We found that the right inferior frontal and thalamic regions, that were engaged by auditory sentence-to-picture matching in Experiment 3 and damaged in patients with auditory sentence-to-picture matching in Experiment 2, were significantly more activated (Z scores = 3.9 and 3.8, respectively) when one-back matching was performed in the absence of semantic information (i.e. for pseudowords and baselines compared to word and object stimuli) (Fig. 2B). There were no significant effects in any part of the right hemisphere that could be attributed to the demands on phonological or semantic processing or the interaction between semantic and phonological processing. In other words, the response in the right hemisphere, including our regions of interest, was more consistent with non-linguistic than linguistic processing demands.

## Discussion

By using a methodological approach that integrates behavioural, lesion and functional imaging data, we argue below that speech comprehension can be impaired after right hemisphere stroke because: (i) normal speech comprehension increases the demands on non-linguistic working memory; and (ii) non-linguistic working memory (an executive function) is supported by right hemisphere regions.

In brief, the behavioural data allowed us to identify a group of patients who had right hemisphere damage and poor scores on one or more language tasks, and generate hypotheses to explain what level of processing impairment (e.g. perceptual, semantic, syntactic or executive) might underlie their poor language scores. The lesion analyses enabled us to create regions of interest by comparing the lesion sites in patients with right hemisphere damage and poor language task scores to the lesion sites in other patients who had right hemisphere damage in the absence of impaired language task scores. Finally, functional imaging allowed us to show that parts of the regions identified in the lesion study were activated when neurologically normal participants performed language tasks and that activation in these regions was more responsive to non-linguistic than linguistic working memory demands. Below, we consider the results of our behavioural, lesion and functional imaging analyses in the context of prior literature in order to demonstrate the scientific novelty, clinical implications and limitations of our findings.

### Behavioural data: the language task and processing level most frequently affected by right hemisphere damage

Previous studies have reported that the incidence of acquired language disorders is ∼1–13% for right-handed right-hemisphere stroke patients (Alexander and Annett, 1996; Coppens *et al.*, 2002) and 18–38% for right-handed left-hemisphere stroke patients (Pedersen *et al.*, 1995). The results from Experiment 1 are consistent with these prior studies but also show how the incidence of language impairments is task-dependent, even after controlling for visual perceptual abilities. For patients with unilateral right hemisphere damage, the highest incidence of impaired performance (13%) was recorded for auditory sentence-to-picture matching, which tests spoken sentence comprehension abilities. This cannot simply be explained in terms of task difficulty, because, in patients with left hemisphere damage, the most frequently impaired task was spoken picture description. Our findings are, therefore, consistent with prior literature in post-stroke aphasia showing that the right hemisphere might contribute to speech comprehension more than speech production (Zaidel, 1976; Crinion and Price, 2005).

By examining how the patients with right hemisphere damage and impaired auditory sentence-to-picture matching performed on other cognitive and language tasks, we identified a group of nine patients who were not impaired on tasks that collectively place similar demands on visual and auditory perception, phonological, semantic and syntactic processing and verbal short-term memory. We therefore hypothesized that their difficulty matching auditory sentences to pictures might be related to instances when word order needed to be held in memory or the task placed high demands on executive (working memory) functions. These types of processing may be more demanding during auditory than visual sentence-to-picture matching, because the auditory sentences are only heard once, before decisions and responses are required, whereas the patient can continue reading the sentence while making a decision with written sentence-to-picture matching. Although it was not possible to assess the patient’s deficits further, we conclude that their difficulties with auditory sentence-to-picture matching were more likely to be the consequence of disrupted executive processing than impairments in linguistic or perceptual processing. It is also possible that mild executive-semantic impairments, paired with disrupted connectivity from auditory input, gives rise to semantic ‘access’ deficits affecting the auditory modality only (Thompson and Jefferies, 2013).

Abnormally low auditory sentence-to-picture matching scores, in the context of good perceptual skills (as observed in our nine patients of interest) are likely to reflect impaired speech comprehension in everyday conversations, even if the patients were not fully aware of their own limitations. This is because the auditory sentence-to-picture matching task includes simple constructions (‘The woman is drinking’) plausibly encountered in everyday speech as well as ones (‘The flower in the cup is blue’) mirrored in everyday relative clauses (‘And the plans that are available to us range from kind of mediocre to really sweet’; cited in Roland *et al.*, 2007).

### Lesion analyses: the right hemisphere lesion sites associated with impaired auditory sentence-to-picture matching

In Experiment 2, we found that the right hemisphere regions that were most frequently damaged in patients with impaired auditory sentence-to-picture matching included dorsal parts of the superior longitudinal fasciculus impinging on the right inferior frontal sulcus, and more ventral subcortical regions in the vicinity of the right putamen, thalamus and caudate (Fig. 1). Damage to these right hemisphere regions was: (i) frequently observed (32% had substantial damage to at least one of these regions); and (ii) not infrequently associated with impaired auditory sentence-to-picture matching (e.g. 67% of those with damage to the VBM region had impaired auditory sentence-to-picture matching when tested months after their stroke). Inter-patient variability in the effects of lesions to regions that show highly significant effects in group-level voxel-based analyses has also been observed in studies of patients with left hemisphere damage. For example, in Gajardo-Vidal *et al.* (2018), we found that the incidence of long-term lexical retrieval impairments following damage to regions identified in group-level voxel-based analyses (with very conservative statistical thresholds) was <50%. If inter-patient variability is due to differences in the ability to recover, future studies should find that variability is less when patients are tested in the acute stage of stroke before recovery from initial deficits occurs. The lesion-deficit association might also be more consistent across subjects if the tests used more sensitive measures of impairments such as reaction times (which are not currently available from our assessments).

Although further studies are required to understand which patients are more versus less affected by right hemisphere damage, the key point here is that the effect of damage to these right hemisphere sites was not atypical. The results of the lesion analyses were therefore used to provide regions of interest for an investigation of how the right hemisphere responds to language and executive processing in neurologically normal participants.

### Functional imaging data: the contribution of the identified right hemisphere regions to normal speech comprehension

Formal evidence that the right hemisphere is normally involved in matching spoken sentences-to-pictures is provided by Experiment 3. Within the regions that were damaged in patients with auditory sentence-to-picture matching impairments, activation was observed in the right inferior frontal sulcus and right mediodorsal thalamus. In addition, these regions responded during a range of language tasks, with no evidence to suggest that they were particularly responsive to perceptual, semantic, phonological or syntactic processing. The results of a second functional imaging experiment (Experiment 4) explain this finding by showing that the right inferior frontal sulcus and right mediodorsal thalamus are sensitive to the demands on non-linguistic (i.e. domain-general) working memory capacity because they were significantly more activated when one-back matching was performed on stimuli that lacked semantic content (Fig. 2B). Our data thus complement and extend the results of previous studies of semantic cognition (Jefferies, 2013; Thompson *et al.*, 2016) that have shown that regions in the right middle cerebral artery territory contribute to executive aspects of semantic processing (i.e. controlled semantic retrieval). Using functional imaging of neurologically normal participants, we show the most critical region is likely to be the right inferior frontal sulcus and that the function of this region is not limited to semantic tasks.

Our neuropsychological, lesion and functional MRI data therefore collectively support the hypothesis that difficulties performing the auditory sentence-to-picture matching task after right hemisphere damage could result from disruption to non-linguistic executive processing that is necessary for normal language function.

### Scientific novelty

Our findings link three unrelated observations in the prior literature: (i) right inferior frontal and right mediodorsal thalamus activity increase during executive processing; (ii) executive processing is required for sentence comprehension; and (iii) right frontal activity increases during sentence comprehension.

The role of the right inferior frontal and right mediodorsal thalamus in executive functions (e.g. planning, monitoring, switching and inhibition) has been demonstrated in many prior studies (Aron *et al.*, 2004, 2014; Halassa and Kastner, 2017; Neef *et al.*, 2018). For example, neuropsychological studies have reported difficulties in working memory and inhibitory control after focal damage to both the right frontal lobe (Szczepanski and Knight, 2014) and the right mediodorsal thalamus (Van der Werf *et al.*, 2003); and multiple functional MRI studies have reported activation changes in right inferior frontal cortex (Sebastian *et al.*, 2016) and right mediodorsal thalamus (Andrews *et al.*, 2006; Minzenberg *et al.*, 2009) under a variety of test conditions that tax executive functions. Evidence that these two regions work as part of a single executive system is further provided by anatomical connectivity studies that have identified reciprocal fronto-thalamic connections (Behrens *et al.*, 2003; Hwang *et al.*, 2010; Eckert *et al.*, 2012; Jeon *et al.*, 2014).

The importance of good executive functions for speech comprehension has been shown by manipulating ambiguity in the sentence content (Key-DeLyria and Altmann, 2016). For example, individuals with higher IQ scores and faster processing were more likely to answer ambiguous sentence comprehension questions correctly (Engelhardt *et al.*, 2017), and older adults with good inhibition skills showed better sentence comprehension than those with poor inhibition skills (Yoon *et al.*, 2015). Interestingly, syntactic interference effects during sentence comprehension were found to be predicted by general working memory capacity but not by phonological memory capacity as measured by digit span (Tan *et al.*, 2017). This highlights a role for non-linguistic working memory in sentence comprehension that is over and above the contribution of verbal working memory capacity and may explain why our patients were not found to have abnormally low digit spans.

The role of the right inferior frontal lobe in sentence comprehension has also been shown previously, particularly for older compared to younger neurologically normal participants (Wingfield and Grossman, 2006), when words are ambiguous (Mason and Just, 2007), when sentences are reversible (Meltzer *et al.*, 2010) or indeterminate (de Almeida *et al.*, 2016), and when patients with aphasia after left hemisphere strokes are recovering their sentence comprehension abilities (van Oers *et al.*, 2010; Mohr *et al.*, 2014; Kielar *et al.*, 2016). Finally, implication that the right hemisphere may be playing a non-linguistic executive role in normal speech comprehension has also been proposed (Bozic *et al.*, 2010; Vigneau *et al.*, 2011; Baumgaertner *et al.*, 2013).

Together, these studies provide abundant evidence that non-linguistic executive processing in the right hemisphere is important for speech comprehension. Nonetheless, we are not dismissing the potential contribution of right hemisphere regions to language processing itself. For instance, there is accumulating evidence showing that bilateral anterior temporal lobes are involved in the representation of conceptual knowledge (Rice *et al.*, 2015; Jung and Lambon Ralph, 2016; Lambon Ralph *et al.*, 2017).

Our study adds to previous literature in several ways. First, we demonstrate that damage to the right inferior frontal sulcus and right mediodorsal thalamus can impair spoken sentence comprehension. Second, we show that the effect of damage to these regions can be explained by disruption to normal functional anatomy. Third, we experimentally link the literature on three unrelated topics (summarized above) by identifying a right inferior frontal region that is (i) damaged in patients who have auditory sentence-to-picture matching impairments; (ii) activated when neurologically normal participants are performing auditory sentence-to-picture matching; and (iii) sensitive to the demands on non-linguistic and linguistic working memory. This provides the first evidence that the same right hemisphere regions are contributing to both sentence comprehension and executive function.

### Clinical relevance

Our study shows that damage to the right inferior frontal cortex and right mediodorsal thalamus can impair sentence comprehension, and this is likely to be the consequence of disruption to a right hemisphere system that is involved in normal language processing. Our conclusion complements findings from patients with semantic dementia, which highlight the role of the right anterior temporal lobe in semantic representations (Rice *et al.*, 2015; Jung and Lambon Ralph, 2016; Thompson *et al.*, 2016; Lambon Ralph *et al.*, 2017). However, we are not claiming that language impairments following right hemisphere strokes can always be explained by a disruption to parts of the normal language system. Other patients may present with impaired language abilities after right hemisphere damage as a result of atypical language lateralization (i.e. crossed aphasia; Marien *et al.*, 2004).

### Limitations, clarifications and future directions

Our study has focused on a carefully selected group of patients who had the most consistent language impairment after right hemisphere stroke and demonstrated that this language impairment can be explained by disruption to normal functional anatomy. However, we are not excluding the possibility that language difficulties in other patients with right hemisphere damage can be a consequence of atypical hemispheric lateralization of language prior to their stroke. Future studies are needed to demonstrate this formally by combining neuropsychological data with functional MRI investigations of neurologically typical participants.

In neurologically normal participants, we identified a right inferior frontal region and a right mediodorsal thalamic region that were both activated during language tasks (Experiment 3) and also when the demands on non-linguistic working memory increased (Experiment 4). Future neuropsychological studies are needed to assess whether damage to these regions impairs non-linguistic (domain-general) executive functions as well as sentence comprehension.

Three of nine patients of interest were not aware that their everyday speech comprehension had been compromised by their stroke. Their lack of awareness is not unexpected given that, in a previous study, we found that patients with comprehension difficulties are less aware of their impairments than patients with speech production difficulties (unpublished results). However, future studies are needed to investigate sentence comprehension and self-awareness of comprehension abilities in more detail and to establish how these measurements change over time post-stroke.

Finally, we note that, although the functional MRI experiments focus on the function of the grey matter regions, we are not dismissing the likely contribution of the surrounding white matter, which is expected to play the important role of propagating activity to and from other task-related regions.

## Conclusion

By combining behavioural, lesion and functional MRI data, our results demonstrate that: (i) long-lasting speech comprehension impairments were frequently observed in our right-handed patients with right inferior frontal damage; (ii) this can be explained by disruption to normal functional anatomy rather than being indicative of crossed aphasia/atypical language lateralization; and (iii) the same right hemisphere regions contribute to both sentence comprehension and executive functions. Seemingly domain-specific cognitive deficits (e.g. language processing) can therefore be explained by disruptions to domain-general cognitive mechanisms (e.g. non-linguistic executive processing). Conversely, these findings are consistent with previous reports that attribute increased right inferior frontal activity during speech comprehension, in patients with left hemisphere lesions, to a greater reliance on domain-general cognitive processing (van Oers *et al.*, 2010; Geranmayeh *et al.*, 2014).

Clinically, our study highlights the possibility that speech comprehension difficulties after right hemisphere stroke may be long-lasting and not due to atypical language lateralization. Given that around 10 million people worldwide survive a stroke each year (Feigin *et al.*, 2017), the number of patients with speech comprehension difficulties after right hemisphere damage is likely to be substantial, in right-handed as well as left-handed patients. Clinical advice should therefore alert patients with right hemisphere stroke to the possibility that they may suffer from long-lasting speech comprehension difficulties, rather than informing them that right hemisphere strokes do not typically cause long-term language difficulties.

## Supplementary Material

Supplementary Data

## Abbreviation

VBM: voxel-based morphometry
