# Supplementary material for: How right hemisphere damage after stroke can impair speech comprehension
Source: Brain. 2018 Nov 9;141(12):3389–404. doi: 10.1093/brain/awy270 (PMC6262220; doi:10.1093/brain/awy270)
Supplement: Supplementary Data [file awy270_suppl_data.pdf]

SUPPLEMENTARY MATERIAL

Supplementary Table 1. Performance of the patients of interest across the 6 cognitive tasks of the CAT

Patient ID	Task 1 Av. score	Task 1	Task 2	Task 3	Memory Score	Task 4	Task 5	Task 6
**1**	0.33	53	48	60	54	69	60	57
**2**	0.00	66	59	60	62	70	60	65
**3**	-0.33	53	43*	60	50	69	55	44
**4**	-0.33	53	59	60	62	72	60	53
**5**	-0.33	53	48	60	54	67	68	57
**6**	0.00	66	48	60	54	71	60	65
**7**	-0.5	48	59	47*	50	75	60	65
**8**	0.16	59	48	51	50	70	68	53
**9**	0.00	66	59	60	62	69	60	65

The table displays for every patient in the group of interest the test scores (i.e. T-scores) on the 6 cognitive tasks of the Comprehensive Aphasia Test. Memory score is a combined score from picture-to-picture semantic matching and recognition memory tasks. * denotes impaired performance.

Task 1 Av. Score = represents the average deviance from the centre of the line for the line bisection task, which provides a quantitative description of normal (-0.5 to +0.5), mild (-1 to +1), or severe (-2 to +2) visual field defects (i.e. neglect). **Task 1** = Line bisection; **Task 2** = Picture-to-picture semantic matching; **Task 3** = Recognition memory; **Task 4** = Word fluency; **Task 5** = Gesture object use; **Task 6** = Arithmetic. Patient Numbers 1-9 refer to the following IDs in the PLORAS database: PS0316, PS0383, PS0448, PS0670, PS0870, PS1172, PS1211, PS1550 and PS2627.

Supplementary Table 2. Performance of the 9 patients of interest on 21 CAT tasks

Task	P1	P2	P3	P4	P5	P6	P7	P8	P9
Match aud word-to-pic	55	55	60	65	60	65	60	65	53
Match aud sentence-to-pic	**57***	**58***	**58***	**58***	**58***	**58***	**58***	**60***	**57***
Match aud paragraphs	60	49	60	60	49	60	60	60	49
Match written word-to-pic	55	59	65	65	65	65	59	**51***	**47***
Match written sentence-to-pic	62	64	62	65	65	64	67	59	62
Repetition of heard words	57	57	57	65	57	57	**56***	57	57
Repetition of complex words	62	62	62	62	62	62	62	62	62
Repetition of nonwords	55	53	67	67	55	53	62	67	67
Repetition of digit strings	55	66	55	66	55	59	55	55	59
Repetition of sentences	63	63	63	63	63	63	63	63	63
Naming objects	**60***	74	66	70	64	74	74	66	**60***
Naming actions	69	**59***	69	69	69	69	69	59*	69
Spoken picture description	66	62	62	72	74	61	66	68	**58***
Reading words	69	62	64	62	69	69	62	**57***	64
Reading complex words	67	67	67	67	67	67	67	**51***	67
Reading function words	62	62	62	62	62	62	62	49	62
Reading nonwords	68	68	61	64	68	68	68	58	68
Copying letters	61	61	61	61	61	61	52	61	61
Written picture naming	67	67	62	67	67	67	58	55	67
Writing to dictation	**57***	63	59	63	61	61	63	**54***	68
Written picture description	68	69	67	75	75	75	68	**64***	70

The table displays for every patient in the group of interest the test scores (i.e. T-scores) on the 21 language tasks of the Comprehensive Aphasia Test. * denotes impaired performance. Aud = auditory stimuli (heard speech); pic = picture.

Supplementary Table 3. The auditory sentence-to-picture matching task.

Sentence Number	Sentence Type	Number of predicates	Example
1, 2, 3	NP	1	The woman is walking
4, 5	NP VP NP A	2	The man is eating the apple
6	NP VP PP	2	The dog is sitting on the table
7	NP VP NP	2	The apple is under the shoe
8, 9, 12	NP VP NP A	2	The singer hits the soldier
10, 11	NP VP NP P	2	The policeman is painted by the dancer
13	NP (*PP) VP NP E	2	The shoe under the pencil is blue
14	NP(*clause) VP NP E	2	The carpet the cat is on is red
15	NP VP PP	2	The red pencil is under the shoe
16	NP (*PP) VP NP	1	The flower in the cup is blue

Abbreviations: NP = noun phrase, VP = verb phrase, PP = prepositional phrase, A = active sentence, P = passive sentence, E = embedded sentence, * = post-modifying. Sentences 1-6 were non-reversible, sentences 7-16 were reversible.

Supplementary Figure 1. Examples of stimuli for each of the 10 conditions from Experiment 3, followed by factorial combinations of these tasks (Designs A, B and C) and the contrasts used to test the effects of interest (abbreviations on next page).

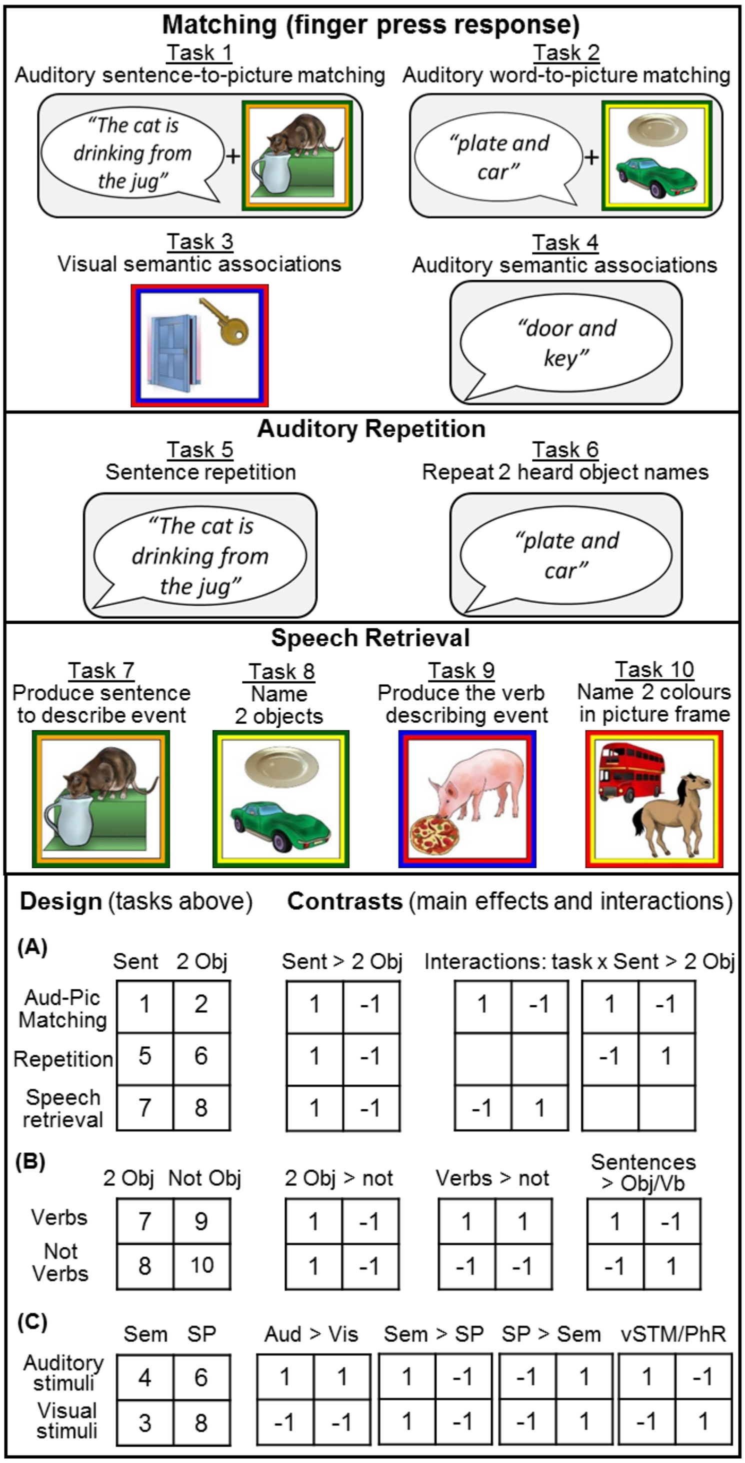

Key to Abbreviations in Supplementary Figure 1

Design A

- Aud-Pic Match = auditory speech-to-picture matching (sentences or 2 object names).
- Repetition = auditory repetition of sentences or 2 object names.
- Speech retrieval = producing a sentence to describe the interaction (event) between 2 objects in a picture or naming 2 unrelated objects.
- Sent = hearing or producing a sentence.
- 2 Obj = hearing 2 unrelated object names or seeing 2 unrelated objects in a picture.

Design B

- Verbs = producing a sentence or a verb that describes the event between 2 objects (e.g. eating).
- Verbs > not = naming verb (or producing sentence) > object and colour naming.
- 2 Obj = naming two objects in a picture or a sentence describing the event between 2 objects.
- 2 Obj > not = naming 2 objects (unrelated or in event) > verb and colour naming.
- Sentences > Obj/Verbs = producing a sentence > naming objects or verbs without sentence.

Design C

- Sem = semantic association of two objects in pictures (visual) or 2 heard object names (auditory).
- SP = speech production (naming objects in pictures, or repeating heard object names).
- Aud = auditory stimuli (e.g. 2 heard object names).
- Vis = visual stimuli (i.e. pictures of 2 unrelated objects).
- vSTM = verbal short-term memory (highest for semantic association on heard object names).
- PhR = phonological retrieval (highest when naming objects from pictures).

Supplementary Figure 2. Example stimuli from the CAT auditory sentence-to-picture matching task (A) and from the fMRI auditory sentence-to-picture matching task (B)

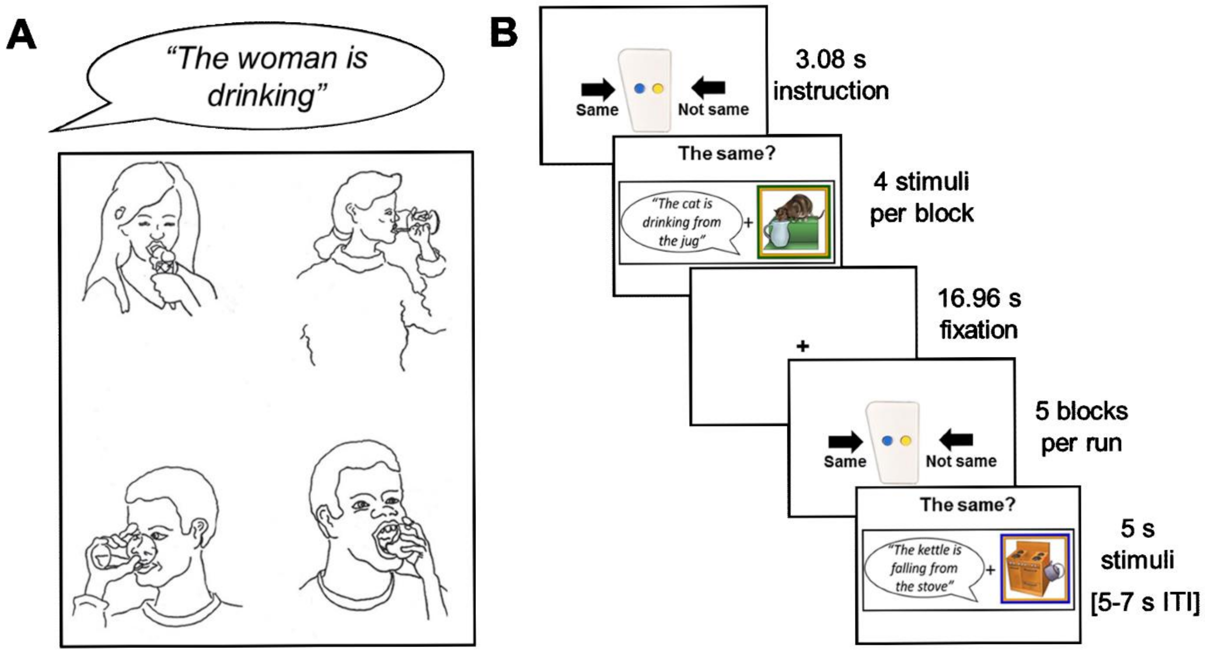

(A) The left panel shows sentence number 1 from the CAT auditory sentence-to-picture matching task. Patients were instructed to hear a sentence (produced by the examiner), and select the picture, from a set of 4, that best illustrates the sentence. The target sentence was "the woman is drinking" (right upper corner). **(B)** The right panel shows the fMRI auditory sentence-to-picture matching task (Experiment 3). Sentences in the bubble were presented aurally.

Supplementary Figure 3. Lesion overlap map of the full sample of 109 right hemisphere stroke patients

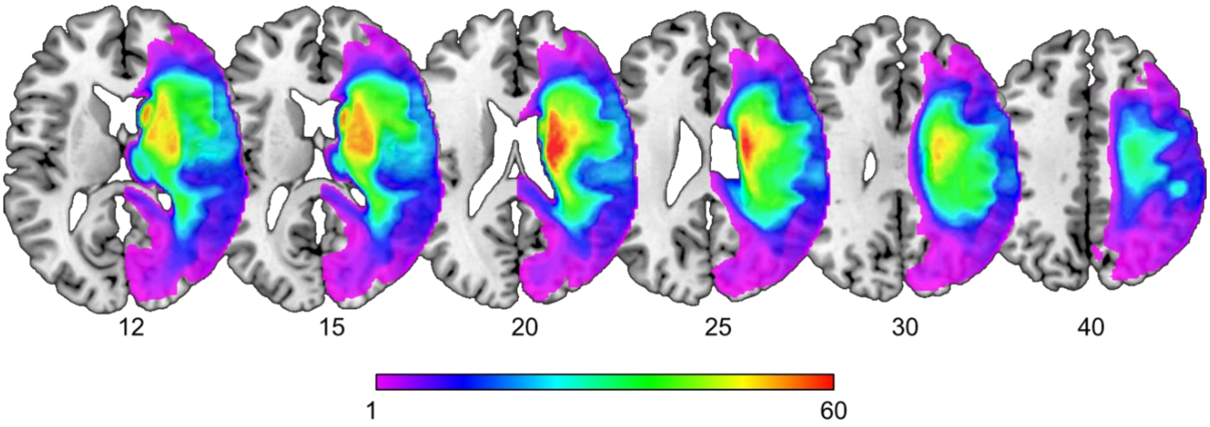

Lesion overlap map for the full sample of 109 stroke patients, depicting voxels that were damaged in a minimum of 1 and a maximum of 60 patients. The colour scale indicates the number of patients with overlapping lesions at each given voxel. The highest frequency of damage (≥ 50% of patients) was observed in the periventricular white matter.

Supplementary Figure 4. Lesion overlap maps (A) and lesion difference map of the patients of interest minus the non-impaired control patients (B).

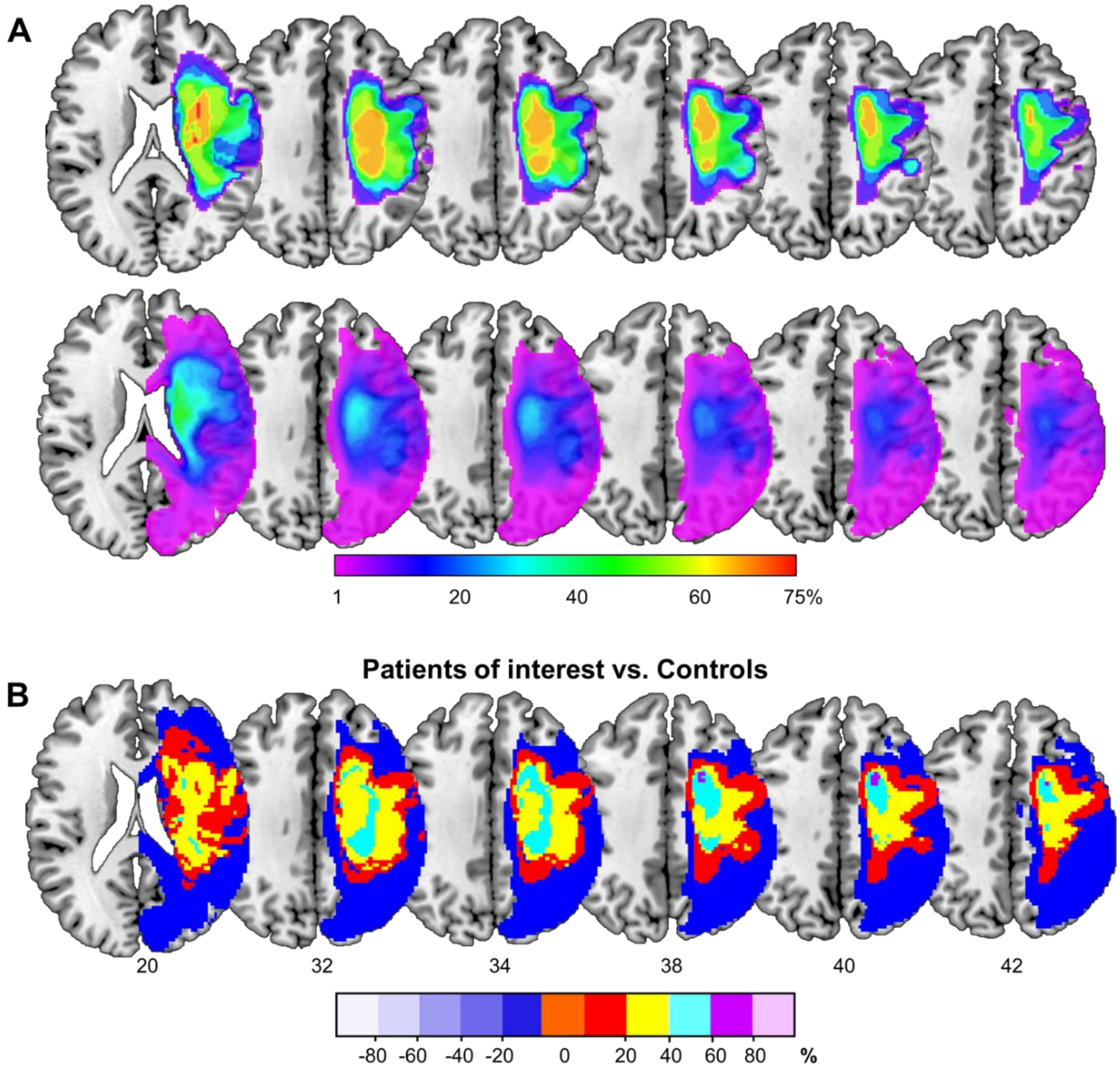

(A) The top and middle rows show the lesion overlap maps for the 9 patients of interest and the 75 non-impaired control patients, respectively. The colour bar on the bottom shows the percentage (0-75%) of patients who have a lesion including that particular voxel. The areas of maximum lesion overlap for the group of interest and the control group were: 7/9 (78%) and 42/75 (56%).

(B) The bottom row shows the lesion difference map for the images shown in part A (Karnath et al., 2004). The five different colours code increasing frequency differences (percent overlap in patients of interest – percent overlap in control patients) in 20% increments: (i) red = difference 1 to 20%; (ii) yellow = difference 21 to 40%; (iii) cyan = difference 41 to 60%; (iv) purple = difference 61 to 80% and (v) light pink = difference 81 to 100%.

References

Karnath H-O, Fruhmann Berger M, Kuker W, Rorden C. The anatomy of spatial neglect based on voxelwise statistical analysis: a study of 140 patients. Cereb Cortex, 2004; 14:1164-72.
